# Quality of Life and Depressive Symptoms Among Peruvian University Students During the COVID-19 Pandemic

**DOI:** 10.3389/fpsyg.2022.781561

**Published:** 2022-02-23

**Authors:** Joel Figueroa-Quiñones, Julio Cjuno, Daniel Machay-Pak, Miguel Ipanaqué-Zapata

**Affiliations:** ^1^Instituto de Investigación, Capacitación y Desarrollo Psicosocial y Educativo: PSYCOPERU, Lima, Peru; ^2^Escuela Profesional de Psicología, Universidad Peruana Unión, Lima, Peru; ^3^Red Latinoamericana de Salud Mental en Pueblos Originarios, Lima, Peru

**Keywords:** quality of life, depressive symptoms, student, university, COVID-19, pandemic, Peru

## Abstract

**Objective:**

To determine the factors associated with quality of life and depressive symptoms in Peruvian university students during the COVID-19 pandemic.

**Methods:**

Multicentre study in 1,634 students recruited by convenience sampling. The quality of life (QoL) was assessed with the European Quality of Life-5 Dimensions at three levels (EQ-5D-3L) and depressive symptoms with the Patient Health Questionnaire-9 (PHQ-9). To assess factors associated with QoL and depressive symptoms, linear regressions and fitted regressions were used, with robust coefficients of variance information (β).

**Results:**

A 345 (21.1%) reported problems in performing daily activities, 544 (33.3%) reported pain and discomfort, 772 (47.2%) were moderately/very anxious or depressed. Furthermore, 207 (12.7%) had moderate-severe and severe depressive symptoms. Men reported better QoL than women (β: 3.2; 95% CI: 1.1, 5.4; *p* = 0.004) and fewer depressive symptoms (β: −0.7; 95% CI: −1.3, −0.2; *p* = 0.011). Ayacucho’s residents had more depressive symptoms than Ancash’s residents (β: 0.8; 95% CI: 0.1, 1.5; *p* = 0.022) and Piura’s residents had fewer depressive symptoms than Ancash’s residents (β: −1.195% CI: −1.8, −0.3, *p* = 0.005). Students who left home during quarantine reported more depressive symptoms (β: 0.7, 95% CI: 0.2, 1.2, *p* = 0.006).

**Conclusion:**

Problems performing daily activities, pain and discomfort, as well as mild to severe depressive symptoms were found in more than three-quarters of the sample. Authorities could consider depression care to improve quality of life in regions where high rates of infection occurred during the pandemic.

## Introduction

Coronavirus disease (COVID-19), classified as a pandemic by the World Health Organization (WHO), is a public health problem that has affected the entire world population. The disease has signs and symptoms similar to those of a common cold, but its complication in at-risk patients can be fatal (World Health Organization, 2020a). In September 2021, it had infected more than 200 million people worldwide and caused more than four million deaths (World Health Organization, 2020b). Latin America has been one of the most affected cities, with more than 40 million cases of COVID-19 and 1,477,000 deaths (Pan American Health Organization, 2020).

Measures to control the spread of COVID-19, such as a state of national emergency and mandatory social isolation (confinement) were declared, have led to changes in people’s life routines that could affect their mental health (Brooks et al., 2020; Rajkumar, 2020). A recent review study in China and Singapore reported the prevalence of anxiety and depressive symptoms in the population in ranges of 6–50% and 14–48%, respectively (Pappa et al., 2020). Factors such as low income, being a woman and being unemployed significantly impair mental health in times of a COVID-19 pandemic (Mejia et al., 2020; Parrado-González, 2020). For example, in low-income countries such as Ethiopia, the prevalence of depressive and anxiety symptoms has been reported to be 46.2 and 48.1%, respectively (Necho et al., 2020). Similarly, the COVID-19 pandemic has hurt the quality of life of these populations. A study with the Bangladeshi residents found that more than 50% of respondents had decreased quality of life, mainly due to difficulties in meeting their basic needs, loss of jobs and barriers to accessing education (Mondal et al., 2021). A study in China found that 41.3% of people had depressive symptoms and a significantly lower quality of life (Ma et al., 2020). In Latin America, there is little literature on assessing the association between quality of life and depressive symptoms in times of pandemic (COVID-19), with only one study in a Brazilian population reporting high levels of depressive symptoms (41.9%) and anxiety symptoms (29.0%), which were associated with poorer quality of life (Vitorino et al., 2021).

In education, the rapid transmission of COVID-19 led to the suspension of face-to-face academic activities, affecting 91.3% of the global student population and 23.4 million higher education students in Latin America and the Caribbean, who had to adapt quickly to non-face-to-face education (Instituto Internacional para la Educación Superior en América Latina y el Caribe, 2020). A review study with more than 436,799 students from the United States and China found stress in 23%, anxiety in 29%, and depression in 37%, so that the abrupt adaptation to virtual education may have deteriorated the quality of life and mental health in this population (Wang et al., 2021). In Bangladesh, a high percentage of university students with depressive (54.5%) and anxious (42.9%) symptoms were reported (Islam et al., 2020). Also, in Europe, there are studies in Greece of 1,000 university students who reported prevalences of 42.5% for anxiety, 74.3% for depression, 63.3% suicidal thoughts, and quality of life has worsened by 43.0% (Kaparounaki et al., 2020). In Italy, a study of 655 university students reported feelings of sadness (51.3%), nervousness (64.6%), irritability (57%), difficulty concentrating (55.9%), difficulty sleeping (54.5%), eating disorders (73.6%), tachycardia (65%), and a tendency to cry (65%) (Commodari et al., 2021).

Peru has been a country most affected by the COVID-19 pandemic. The confinement affected the education of Peruvian students due to economic hardship and the digital divide (internet connectivity and computer use). This action led the government to implement economic policies for Peruvian families and the education sector, such as the issuance of economic bonds, educational credits, connectivity, and scholarships (Figallo et al., 2020; Contraloría General de la República, 2021). For example, the National Institute of Statistics and Informatics revealed that during 2020, monetary poverty of Peruvian households amounted to 30.1%, increasing 10 percentage points from the previous year, affecting mainly 45.7% of the rural population and 26.0% of the urban population. Moreover, 85.7 and 82% of these poor households do not have a computer or Internet access, respectively (Instituto Nacional de Estadística e Informática, 2021), there are only studies before the COVID-19 pandemic in Peru where they reported low levels of quality of life and the presence of depressive symptoms in 52.2 and 24.6% of the university population, respectively (Kuong and Concha, 2017; Diaz-Godiño et al., 2019).

Given the above, there is little research in Latin America and non-existent in Peru on the evaluation of the quality of life (QoL) situation associated with depressive symptomatology and the associated factors of the same, in a population of university students in times of COVID-19, considering that the current pandemic situation generates a greater state of vulnerability (Puthran et al., 2016; Ribeiro et al., 2018), making it difficult to implement programmes and interventions to improve mental health and quality of life in the face of such a state of emergency (Figallo et al., 2020). Therefore, this study determined the factors associated with quality of life and depressive symptomatology in Peruvian university students during COVID-19.

## Materials and Methods

### Study Design

We conducted a multicentre cross-sectional study of students at a private university in four regions of Peru (Ancash, Ayacucho, Lima, and Piura), with large student populations and entrenched socio-cultural differences between July and August 2020.

### Participants

The study participants were 1,634 university students from four regions of Peru (Ancash, Ayacucho, Lima, and Piura), which were obtained from a non-probabilistic convenience sampling, with an online survey sent through social networks (WhatsApp and email), where informed consent was presented through the presentation of the problem and the objective of the study, to subsequently decide the option to voluntarily participate in filling out the questionnaire; those who agreed to participate in the study answered all the questions of the data collection instrument. The eligibility criteria for a student to participate in the study were as follows: (i) Being over 18 years of age, (ii) Residing in the pandemic crisis within the regions of Peru, where the study was conducted, and (iii) Students studying at the undergraduate level within the private university where the data were collected. On the other hand, the only exclusion criterion was to exclude participants who did not fully answer the questions in the questionnaires. However, it is important to mention that it was impossible to take into account the evaluation of previous psychiatric comorbidities, because at the time of data collection, it was not feasible in Peru due to the restrictions established.

### Procedures

The electronic survey generated for the study followed the quality improvement recommendations for web-based surveys based on the Checklist for reporting results of Internet e-surveys (CHERRIES) (Eysenbach, 2012).

The development and data collection of the electronic instrument was carried out through the Survey Monkey virtual platform (Survey Monkey, 1999). Initially, this electronic instrument consisted of an introduction explaining the composition of the work team, the objectives of the study, anonymity, confidentiality of the data and the use of the information for scientific purposes only. Subsequently, students were given informed consent to continue with the survey. The time to complete the questionnaire was 15 min.

The survey was promoted through emails and university student study groups (WhatsApp), between the period of July and August 2020. Participants were free to opt out of the questionnaire at any time, without explanation, and were not asked to identify themselves due to the confidentiality of the information. Finally, all the surveys completed by university students were securely stored using a password-protected database.

### Variables

The primary variables were quality of life (QoL) and depressive symptomatology in university students from four regions of Peru. The QoL variable was measured through the European Quality of Life-5 Dimensions questionnaire in three levels (EQ-5D-3L) (EuroQol, 2017), composed of five items that respond to five dimensions (Mobility, Self-care, Daily activities, Pain or Discomfort, Depression or Anxiety), with three levels of response (absence, moderate presence, and severe presence). It also has a visual analog scale (EQ-VAS) that reports current life status on a range from 0 (worst status) to 100 (best status), this last indicator of QoL. We used the cultural adaptation of the EQ-5D-3L translated into Spanish available for Peru, carried out by the EuroQol Group, which was responsible for the original instrument and its theoretical validation (Herdman et al., 2003). The EQ-5D-3L is a widely used instrument worldwide to assess quality of life. Some studies have conducted psychometric validity of this instrument, reporting adequate indicators of discriminant validity (Shannon’s H′: 0.47 and 0.98) and good reliability (weighted Kappa _w_K: 0.39–0.93); however, this type of validation was performed on the original version (Buchholz et al., 2018). On the other hand, there are few psychometric validation studies in the Spanish-speaking version using the general and/or clinical population (García-Gordillo et al., 2015). In Peru, this type of validation has not yet been conducted; however, several studies in the country report similar and consistent results within this geographical context (Taype-Rondan et al., 2017; Figueroa-Quiñones et al., 2019).

The Patient Health Questionnaire-9 (PHQ-9) was used for the variable depressive symptoms. It is a nine-item self-administered questionnaire with five response levels according to the frequency of depressive symptoms in the last 2 weeks: not at all, several days, more than half of the days, and almost every day. The total score is within the range of 0 to 27 points. Depressive symptoms are also reported according to severity levels: minimal (0–4), mild (5–9), moderate (10–14), moderate-severe (15–19), and severe (20–27) (Spitzer et al., 1999; Kroenke et al., 2001; Cameron et al., 2008). This questionnaire has been validated in the Peruvian population, presenting indicators of psychometric properties through confirmatory factor analysis (CFI = 0.936, TLI = 0.914, RMSEA = 0.089 and SRMR = 0.039) and reliability of Cronbach’s Omega and Alpha indicators (ω = 0.87 and α = 0.87), interpreted in such a way because many indicators such as CFI and TLI are close to 0.90; the RMSEA at 0.08 and the SMRM is lower than 0.08; while, for reliability, the values of Cronbach’s omega and alpha indexes are higher than the optimal point (0.80) (Villarreal-Zegarra et al., 2019).

The covariates that participated in the study were age (in tertiles), sex (male or female), regions of residence (Ancash, Ayacucho, Lima, and Piura), marital status (single, separated, widowed, divorced, and married or partner), occupation (studying and working or only working), left home during quarantine (no and yes), decrease in family income in quarantine (no and yes), lives alone (no and yes) and family with chronic disease (no and yes).

### Data Analysis

A descriptive analysis was presented using measures of central tendency and dispersion (for numerical variables) and absolute frequencies (for categorical variables). We performed linear regression with robust variance reporting crude, adjusted model with coefficients (β) and their 95% confidence intervals (CI95%) to evaluate the association of the factors associated with the EQ-VAS (quality of life) and PHQ-9 (depressive symptoms) scores. In all cases, variables that obtained a *p* < 0.20 in the crude model were included in the adjusted model. Analyses were performed in Stata v15.0 statistical software (StataCorp, 2017).

### Ethics Statement

This study was reviewed and approved by the Ethics Committee of the Universidad Católica Los Ángeles de Chimbote (Los Angeles de Chimbote Catholic University). In addition, the study was anonymous and voluntary, so it does not pose a risk to participants who accepted their participation with the online-informed consent. With the approval of the ethics committee and the ethical steps followed for data collection, we sought to ensure compliance with the National Commission for the Protection of Human Subjects of Biomedical and behavioral Research (Commission for Protection of Human Subjects of Biomedical and Behavioral Research, 1978).

## Results

The 1,825 participants were initially recruited, of which 65 did not agree to participate in the study and 126 did not complete the entire survey; therefore, only 1,634 (89.5%) university students participated in the study. The participants were distributed according to the region of residence: 712 (43.6%) are from Ayacucho, 347 (21.2%) from Ancash, 342 (20.9%) from Piura and, 233 (14.3%) from Lima (Table 1).

**TABLE 1:** Characteristics of the populations studied.

Variables	Total (*n* = 1 634)	Ancash (*n* = 347)	Ayacucho (*n* = 712)	Lima (*n* = 233)	Piura (*n* = 342)
Age: Median (IQR)	24 (20–30)	22 (20–27)	24 (21–30)	22 (19–28)	30 (21–42)
**Age in tertiles (years)**					
16–21	575 (35.2)	151 (43.5)	198 (27.8)	66 (28.3)	160 (46.8)
22–27	524 (32.1)	110 (31.7)	282 (39.6)	36 (15.5)	96 (28.1)
28–65	535 (32.7)	86 (24.8)	232 (32.6)	131 (56.2)	86 (25.1)
**Sex**					
Female	1,146 (70.1)	259 (74.6)	491 (69.0)	145 (62.2)	251 (73.4)
Male	488 (29.9)	88 (25.4)	221 (31.0)	88 (37.8)	91 (26.6)
**Marital status**					
Singer/separate/widowed/divorced	1,270 (77.7)	282 (81.3)	555 (77.9)	156 (66.9)	277 (81.0)
Married/partner	364 (22.3)	65 (18.7)	157 (22.1)	77 (33.1)	65 (19.0)
**Occupation**					
Study and work	842 (51.5)	153 (44.1)	392 (55.1)	146 (62.7)	151 (44.2)
Only study	792 (48.5)	193 (55.9)	320 (44.9)	87 (37.3)	191 (55.8)
**Left home in quarantine**					
No	755 (46.2)	120 (34.6)	332 (46.6)	113 (48.5)	190 (55.6)
Yes	879 (53.8)	227 (65.4)	380 (53.4)	120 (51.5)	152 (44.4)
**Decreasing family financial income in quarantine**					
No	123 (7.5)	25 (7.2)	39 (5.5)	27 (11.6)	32 (9.4)
Yes	1,511 (92.5)	322 (92.8)	673 (94.5)	206 (88.4)	310 (90.6)
Lives alone					
No	1,502 (91.9)	317 (91.3)	636 (89.3)	214 (91.9)	335 (97.9)
Yes	132 (8.1)	30 (8.7)	76 (10.7)	19 (8.1)	7 (2.1)
**Family member with disease**					
No	959 (58.7)	197 (56.8)	461 (64.8)	116 (49.8)	185 (54.1)
Yes	675 (41.3)	150 (43.2)	251 (35.2)	117 (50.2)	157 (45.9)

*IQR, interquartile range.*

Participants had a median age of 24 years (interquartile range: 20–30 years), 1,146 (70.1%) were women, 1,270 (77.7%) reported being single, separated, widowed, or divorced. University students studying and working at the same time were 842 (51.5%) with a significant prevalence in Lima. 879 (53.8%) of the university students reported leaving home during quarantine, 1,511 (92.5%) had a decrease in family income, 1,502 (91.9%) declared not living alone and 959 (58.69%) had a family member with a chronic disease (Table 1).

On the quality of life during the pandemic, 345 (21.1%) reported problems in carrying out daily activities, 544 (33.3%) reported pain and discomfort and 667 (40.8%) reported being moderately anxious or depressed and 105 (6.4%) reported being severe anxious and depressed (Table 2).

**TABLE 2:** Quality of life in Peruvian university students during the COVID-19 pandemic.

Quality of life	n (%)
**Mobility**	
I have no problems walking	1,526 (93.4)
I have some problems walking	91 (5.6)
I have to be in bed	17 (1.0)
**Personal care**	
I have no problems with the personal care	1,558 (95.4)
I have some problems washing myself	69 (4.2)
I’m unable to wash or dress myself	7 (0.4)
**Daily activity**	
I have no problems in performing my activities	1,284 (78.6)
I have some problems to performing my activities	345 (21.1)
I’m unable to performing my activities.	5 (0.3)
**Pain or discomfort**	
I have no pain or discomfort	1,052 (64.4)
I have moderate pain or discomfort	544 (33.3)
I have a lot of pain or discomfort	38 (2.3)
**Anxiety or depression**	
I’m not anxious or depressed	862 (52.8)
I’m moderately anxious or depressed	667 (40.8)
I’m very anxious or depressed	105 (6.4)
**EQ-VAS total**	
Median (SD)	76.0 (25.6)

*SD, standard deviation.*

In addition, 741 (45.4%) students had mild and moderate depressive symptoms, while 207 (12.7%) had moderate-severe and severe depressive symptoms (Table 3).

**TABLE 3:** Depressive symptoms in Peruvian university students during the COVID-19 pandemic.

Depressive symptoms	n (%)
**Depression**	
Minimum	686 (42.0)
Mild	475 (29.1)
Moderate	266 (16.3)
Moderate-severe	132 (8.1)
Severe	75 (4.59)

The univariate analysis reported that during the COVID-19 pandemic, being male and residing in Lima or Piura were associated with higher EQ-VAS (quality of life) scores; whereas, being between 22 and 27 years of age, is dedicated only to study, residing in the Ayacucho region, leaving home during quarantine, suffering a decrease in family income during quarantine, having a family member with chronic illness and depressive symptom scores decreased QoL scores (Table 4).

**TABLE 4:** Factors associated with quality of life and depressive symptoms in Peruvian university students during the COVID-19 pandemic.

Variables	Quality of life				Depressive symptoms			
	Crude	p	Full	p	Crude	p	Full	p
	β (IC95%)		β (IC95%)		β (IC95%)		β (IC95%)	
**Age**								
16–21	REF				REF			
22–27	−3.4 (−6.5; −0.3)	0.032	−2.3 (−5.0; 0.4)	0.101	0.51 (−0.2; 1.2)	0.170	−0.1 (−0.6; 0.6)	0.986
28–65	−0.29 (−3.2; 2.6)	0.846	−2.0 (−4.8; 0.8)	0.164	−0.76 (−1.5; −0.1)	0.034	−0.6 (−1.3; 0.2)	0.120
**Sex**								
Female	REF				REF			
Male	7.0 (4.5; 9.4)	0.000	3.2 (1.1; 5.4)	0.004	−1.7 (−2.3; −1.1)	0.000	−0.7 (−1.3; −0.2)	0.011
**Marital status**								
Singer/separated/widowed/divorced	REF				REF			
Married/partner	1.7 (−1.2; 4.5)	0.239	–	–	−1.1 (−1.7; −0.4)	0.002	−0.5 (−1.2; 0.1)	0.112
**Occupation**								
Study and work	REF				REF			
Only study	−2.0 (−4.5; 0.5)	0.118	−0.7 (−3.0; 1.7)	0.565	0.8 (0.2; 1.4)	0.006	0.4 (−0.1; 0.9)	0.145
**Places of residence**								
Ancash	REF				REF			
Ayacucho	−3.5 (−6.8; −0.2)	0.040	−2.1 (−4.8; 0.8)	0.151	0.9 (0.1; 1.7)	0.039	0.8 (0.1; 1.5)	0.022
Lima	4.0 (0.1; 7.9)	0.043	0.6 (−2.8; 4.1)	0.719	−1.6 (−2.5; −0.6)	0.001	−0.7 (−1.5; 0.2)	0.116
Piura	3.6 (0.0; 7.3)	0.049	0.1 (−3.0; 3.3)	0.933	−1.6 (−2.5; −0.8)	0.000	−1.1 (−1.8; −0.3)	0.005
**Went out of the house during quarantine**								
No	REF				REF			
Yes	−3.2 (−5.7; −0.7)	0.012	−0.9 (−3.1; 1.2)	0.396	1.1 (0.5; 1.7)	0.000	0.7 (0.2; 1.2)	0.006
**Decreasing family financial income in quarantine**								
No	REF				REF			
Yes	−8.1 (−11.6; −4.6)	0.000	−3.4 (−6.5; −0.3)	0.034	1.9 (0.8; 2.9)	0.000	0.7 (−0.2; 1.6)	0.141
**Lives alone**								
No	REF				REF			
Yes	−2.1 (−7.1; 3.0)	0.420	–	–	0.8 (−0.4; 2.0)	0.179	0.5 (−0.6; 1.5)	0.383
**Family member with chronic disease**								
No	REF				REF			
Yes	−8.7 (−11.2; −6.1)	0.000	3.7 (−6.1; 1.4)	0.002	2.4 (1.8 – 3.0)	0.000	1.5 (1.0; 2.1)	0.000
**Depressives symptoms^+^/quality of life^++^**	−2.1 (−2.3; −1.9)	0.000	−2.0 (−2.2; −1.8)	0.000	−0.1 (−0.2; −0.1)	0.000	−0.1 (−0.2; −0.1)	0.000

*^+^Depressives Symptoms was obtained from the Patient Health Questionnaire-9 (PHQ-9) total score. ^++^Quality of life was obtained from the Visual Analog Scale (EQ-VAS).*

In multivariate analysis, men reported better quality of life than women (β: 3.2; 95% CI: 1.1, 5.4; *p* = 0.004), and men also had fewer depressive symptoms (β:−0.7; 95% CI: −1.3, −0.2; *p* = 0.011). Those residing in Ayacucho had more depressive symptoms than those residing in Ancash (β: 0.8; 95% CI: 0.1, 1.5; *p* = 0.022) and those residing in Piura had fewer depressive symptoms than those residing in Ancash (β:−1.1; 95% CI: −1.8, −0.3; *p* = 0.005) and, finally, students who left home during quarantine had more depressive symptoms than those who did not (β: 0.7; 95% CI: 0.2; 1.2; *p* = 0.006) (Table 4).

On the other hand, factors associated with PHQ-9 scores (depressive symptoms) reported that all variables were significantly associated. However, after adjusting the model with all variables, we were find that these were still significant: residing in the region of Ayacucho, leaving home in quarantine, and having a family member with chronic disease have higher scores for depressive symptoms; while being male, residing in the region of Piura and higher QoL score decrease scores for depressive symptoms. Our positive relationship results concerning depressive symptom scores show that university students residing in Ayacucho have higher scores compared to those residing in Ancash (β = 0.8, 95% CI = 0.1 to 1.5), those residing in Ancash (β = 0.8, 95% CI = 0.1 to 1.5) and those residing in Piura (β = 0.8, 95% CI = 0.1 to 1.5), those who were quarantined compared to those who were not (β = 0.7, 95% CI = 0.2 to 1.2) and having a family member with a chronic illness presented higher scores in depressive symptomatology compared to those who did not have family members with an illness (β = 1.5, 95% CI = 1.0 to 2.1); the latter factor being the biggest problem. Meanwhile, concerning the negative relationship, men had lower scores than women (β = −0.7, 95% CI = −1.3 to −0.2), with residents of the region of Piura having lower scores than those residing in Ancash (β = −1.1, 95% CI = −1.8 to −0.3). Finally, the quality of life scores decreased by 1 quality of life points increased by 0.1 points in depressive symptoms (β = −0.1, 95% CI = −0.2 to −0.1) (Table 4).

## Discussion

University students in relation to QoL during the pandemic reported some problems in performing daily activities 345 (21.1%), pain and discomfort in 544 (33.3%) and reported being moderately anxious or depressed in 667 (40.8%) and severe anxious and depressed in 105 (6.4%). Furthermore, the PHQ-9 reported that 741 (45.4%) had mild and moderate depressive symptoms, while 207 (12.7%) had moderate-severe and severe depressive symptoms. These results are consistent with the study conducted in Vietnamese university students during the COVID-19 pandemic, which reported greater impairment in the anxiety/depression and pain/discomfort dimensions of QoL (Tran et al., 2020). Another study with young Chinese adults reported similar impairment in QoL dimensions during the pandemic (Ping et al., 2020). These findings may possibly be due to bereavement over the death of students’ family members, isolation and reduced physical activities (Hamid and Jahangir, 2020) and fear caused by overexposure to the media and the high lethality of the virus (Mejia et al., 2020), which may have increased symptoms of anxiety or depression. In addition, the long months of confinement resulted in constant exposure to stress and often manifested in pain and discomfort (Esquivel-Acevedo et al., 2020). On the other hand, non-face-to-face education meant that the student had to sit in front of the computer for a long time, choosing postures that provided comfort, however, incorrect body postures could have generated pain and tension (Yang et al., 2020).

On the other hand, males reported better QoL than females (β: 3.2; 95% CI: 1.1, 5.4; *p* = 0.004) and fewer depressive symptoms (β:−0.7; 95% CI: −1.3, −0.2; *p* = 0.011). This result could be explained by several factors; for example, women nowadays have more responsibilities in the work environment and during confinement the with family support needs (e.g., family caregivers) has increased, leading to more stress, and depression (Verma and Mishra, 2020; Wang et al., 2020). Moreover, confinement as a measure to prevent the spread of COVID-19 and the inability to interact socially with peers has a negative impact on mental health (Brooks et al., 2020; Palgi et al., 2020). Likewise, the new normality that was accompanied by sedentary behavior adopted by students due to non-face-to-face education and financial insecurity for educational expenses may have increased depressive symptoms in university students (Huckins et al., 2020; Islam et al., 2020), as well as; it may have been a reaction associated with confinement and habit changes (Vásquez et al., 2020).

Residents in Ayacucho presented greater depressive symptomatology than those in Ancash (β: 0.8; 95% CI: 0.1; 1.5; *p* = 0.022) and residents in Piura had less depressive symptomatology than those in Ancash (β:−1.1 95% CI: −1.8; −0.3; *p* = 0.005). This result could be explained by the fact that during the evaluation months of our study, the rate of COVID-19 infections and deaths in Piura had decreased, while in Ayacucho, in rural Peru, it was at its highest peak (Plataforma digital única del Estado Peruano, 2020).

In addition, students who left home during the quarantine were find to have greater depressive symptoms than those who were compliant with staying at home (β: 0.7; 95% CI: 0.2, 1.2; *p* = 0.006). Possibly a reason why university students were forced to leave home was the loss of family income, which implies difficulties in accessing treatment, medication and living expenses, affecting the quality of life and leading to the onset of some depressive symptoms (Kretchy et al., 2020; Ping et al., 2020; Tran et al., 2020). A study in China with university students also reported mental health problems due to COVID-19 (Wang and Zhao, 2020). It is important to note that other studies show that these mental health problems are more prominent in students within the adolescent stage (Commodari and La Rosa, 2020) and most likely the entire increase is due to the COVID-19 pandemic; however, the extent of influence of the changes themselves has so far not been determined (i.e., mood and mental impairment) o of this stage in mental health reports (Commodari and La Rosa, 2020). Another reason may have been that this age group, according to case-fatality reports, had a lower mortality risk than adults and the elderly, but the fear of bringing the virus home and infecting their family members may have generated the depressive symptoms (Figueroa-Quiñones and Ipanaqué-Neyra, 2020; Johnson et al., 2020). Depressive symptoms influence and impact the quality of life of university students (Gan and Yuen Ling, 2019).

The strength of our study is that it is the first study to report up-to-date evidence on the quality of life and mental health status of university students in Peru after the confinement of the COVID-19 pandemic. However, the study has some limitations. Due to the confinement by COVID-19, is the non-probabilistic nature of the sampling, which affects the representativeness of the study sample and the probable impossibility of generalizing the results obtained; however, a significant sample size was achieved in different cities of Peru, which produces consistent evidence of university students, and the results obtained were similar to other studies conducted (Chang et al., 2020; Tran et al., 2020). Another limitation of the study was that it did not take into account the inclusion of any restrictions on particular clinical characteristics, the subjects may have had previous psychiatric comorbidities, so the problems found may possibly be slightly inaccurate, and it is recommended that future studies include previous history or mental health treatment. Likewise, the instrument used (EQ-5D-3L) to assess psychometric quality of life was not validated in Peru, however, this instrument has been used in other studies and populations in Peru (Taype-Rondan et al., 2017; Figueroa-Quiñones et al., 2019), moreover, it was translated into Spanish by the EuroQol Group and has been adapted in other countries and languages (EuroQol, 2017).

## Conclusion

It is concluded that university students reported some problems in performing daily activities, pain and discomfort, as well as mild to severe depressive symptoms in more than three quarters of the sample in relation to their QoL during the pandemic. Therefore, our health authorities also consider psychological interventions to reduce depressive symptoms and improve QoL. On the other hand, men reported better QoL than women and lower depressive symptoms. The female students would be a priority in the Peruvian university population for managing depressive symptoms. In addition, Ayacucho’s residents had higher depressive symptoms than Ancash’s residents and Piura’s residents reported lower depressive symptoms than Ancash’s residents. Health authorities might therefore consider addressing depression in pandemics in settings with high infection rates of infection are reported. A clear example is the professional associations of psychology in the cities of Peru most affected by depression should work in coordination with the health and university sectors to design plans and actions for prevention, detection and subsequent emotional support for their students with depressive symptomatology through virtual platforms (electronic devices, hotlines and internet) and to be able to provide immediate professional help to students affected during COVID-19.

It has also been found that students who left home during quarantine had more depressive symptoms than those who did not. Therefore, education authorities also provide contingency plans for students in pandemic situations, as the lack of resources often forces them to leave home. Universities in Peru could mitigate such depressive symptoms in students by generating a more effective promotion of care programmes with a psychological specialist, while it is true that such attention promotion exists, the impact on students is mostly low. The increase in student scholarships and the reduction of pension costs by universities and/or subsidies and economic bonuses for families by the government could also help reduce students’ worries about paying for their studies, considering that in an undeveloped country, many university students must study and work simultaneously to survive and achieve their academic goals.

Furthermore, suicidal tendencies are prevalent behavior in subjects with anxiety/depression problems and are often complicated by underlying pathophysiological factors and due to our findings in the current context by COVID-19 we believe that it might increase the risk for subjects’ mental health and quality of life. Therefore, mental health authorities should plan assessments, interventions and treatments in clinical practice for affected students.

## Data Availability Statement

The datasets presented in this study can be found in online repositories. The names of the repository/repositories and accession number(s) can be found below: https://doi.org/10.7910/DVN/93WCEZ.

## Ethics Statement

The studies involving human participants were reviewed and approved by the Research Ethics Committee of Los Angeles Catholic University of Chimbote. The participants provided their written informed consent to participate in this study.

## Author Contributions

JF-Q carried out the administration of the manuscript. MI-Z performed the methodology, data retention, formal analysis, and supervision of the manuscript. JF-Q and MI-Z were responsible for the visualization and validation of the manuscript. JC was responsible for the acquisition of funds for the manuscript. JF-Q, JC, DM-P, and MI-Z were responsible for the preparation of the first report and the final version of the original manuscript. All authors contributed to the article and approved the submitted version.

## Conflict of Interest

The authors declare that the research was conducted in the absence of any commercial or financial relationships that could be construed as a potential conflict of interest.

## Publisher’s Note

All claims expressed in this article are solely those of the authors and do not necessarily represent those of their affiliated organizations, or those of the publisher, the editors and the reviewers. Any product that may be evaluated in this article, or claim that may be made by its manufacturer, is not guaranteed or endorsed by the publisher.
